# The Effect of Different Rejection Letters on Applicants’ Reactions

**DOI:** 10.3390/bs9100102

**Published:** 2019-09-20

**Authors:** Michela Cortini, Teresa Galanti, Massimiliano Barattucci

**Affiliations:** 1 Faculty of Psychology, University “G. d’Annunzio”, 66100 Chieti, Italy; teresa.galanti@unich.it; 2 Faculty of Psychology, e-Campus University, 22060 Novedrate, Italy; massimiliano.barattucci@uniecampus.it

**Keywords:** selection, fairness, communication

## Abstract

Organisations appear to pay little attention to rejection letters, considered a special form of organisational communication, despite a growing body of literature that shows they play an important role in terms of employer branding. This study aims to empirically test how applicants’ perceptions are affected by differently manipulated rejection letters. In detail, a sample of 138 rejected candidates filled in an ad hoc questionnaire on perceived selection procedure fairness and satisfaction, after receiving a rejection letter where we had manipulated time latency, the politeness formula and customisation. Results suggest that providing a timely, customised and informal notification is something agreeable, which is able to affect, above all, fairness perceptions and intention to re-apply. In detail, the time latency in giving feedback appears to affect the relationship between fairness perception and organisational recommendation and acts more as a mediator rather than an antecedent variable. Considering that providing feedback is a relatively low-cost activity that at the same time has a big impact on job applicants, our results show that organisations should be sensitive to negative feedback communication, especially in relation to response time, in order to support their employer branding.

## 1. Introduction

In a market characterized by labour shortage and frequent turnover, on one side there is a undoubtedly hard contention among candidates for wanted positions, but on the other, competition for an application can be rather accidental. 

The concept of career has lost the connotations of an organisational process and has become a path managed entirely by the individual [1], conditioned daily by occupational circumstances requiring greater flexibility from the worker and the ability to handle multiple identities and roles [2]. In this complicated job search scenario, companies are required to manage recruiting and communication strategies in order to attract and retain the best candidates. Improving external communication related to selection processes becomes strategically important because of the presence of questionable selection practices, significant transformations in HR demand and requests, and temporal discontinuity of jobs and careers [3]: From an application point of view, a good selection communication process can result in cost savings related to the hiring process (through acceptance decisions), corporate image (through recommendations), and administrative workload [4]. Job applicants take into account both explicit and implicit communication they receive from the organisation during the selection procedure to develop their personal view about the organization itself [5]. Contributions in literature show that the levels of positivity and negativity of selection outcome letters (of both acceptance and rejection) have an impact on both the self-image and company image and on reported future intentions of potential recruits [6,7]; consequently, it seems clear that feedback letters concerning the outcome of the selection should not be managed as simply neutral transmitters of employment decisions, and therefore organisations should pay close attention to the way they communicate rejection in order to avoid damage to the corporate image. Research on communication strategies of the outcome of selection that are able to positively modify applicants’ reactions is of fundamental importance to provide indications to HR management on how to better succeed in the feedback process of personnel selection. 

## 2. Feedback in Selection Processes: A Form of Organizational Communication

Framing the relationship between job applicants and organization within the pragmatics of organizational communication [8,9], a candidature for a vacancy should be considered as a relational act between the two actors: Sending a CV is a communicative move which requires it to be followed by a specific communicative action, such as for any adjacency pairs (e.g., questions and answers). As stressed by several authors [10,11,12], a lot of times organizations do not transfer notification, even if this dramatically becomes a way to communicate that has a severe impact in terms of perception. Above all, a notification has to be seen with all the characteristics required by the ongoing interaction, so that, for example, it should be sent on time as possible, and with the appropriate wording; when the feedback is communicated with high delay or the content of the letter is perceived as not appropriate, the candidate constructs meaning, with consequences in terms of Employer Branding [13]. 

According to the Selection Fairness Theory [14], applicant reactions are partially determined by their perception of fairness experienced during the whole selection procedure. Referring to this theoretical framework, the study aims to verify literature indications concerning the relationships between fairness perception and other applicants’ reactions, and, moreover, to analyse the effect of the manipulation of specific characteristics of the communication process (both content and procedural) on candidate perceptions. 

Through its results, the present study tries to provide useful suggestions for the management of relevant aspects to selection practice and procedure, such as the right latency for sending selection feedback, or the specific content characteristics of a refusal letter.

## 3. Applicant Reactions to the Selection Process

Applicant reactions—attitudes, affect, or cognitions an individual might have about the hiring process [11]—have gained momentum in international literature on personnel selection. Selection procedure perceptions have an effect on employer branding, so maintaining a positive image is crucial for employers during the whole selection process from recruitment to the organisational socialisation of newcomers, in order to reduce the risk of losing top candidates [15,16]. 

According to recent meta-analysis [17,18], there are many reasons to study applicants’ reactions. First, applicants who perceive the potential employer as invasive may view the company as less attractive; second, candidates with negative reactions may influence other potential candidates, as word-of-mouth becomes ever more salient in the Web 4.0 era we are living in; third, candidates who perceive selection practices as unfavourable are less likely to accept an employment offer [19]; fourth, candidates who perceived the selection process as unfair may pursue legal complaints and judicial challenges; finally, negative reactions during selection may lead to an applicant being less likely to reapply to a rejecting employer in the future or even to boycotting its products. 

Empirical research on applicant behaviours has been mostly framed within both Organisational Justice Theory and Psychological Contract Theory [10,20]. Organizational justice is a concept developed by Greenberg [21] in order to explain employee attitudes and behaviours as reactions to organizational behaviour and it is focused on fairness perceptions. Employees evaluate the fairness of the outcomes they receive from the organization (distributive justice), of the formal processes by which outcomes are allocated (procedural justice), and of the interpersonal treatment they receive (interactional justice). 

On the other hand, the psychological contract refers to a combination of explicit and implicit obligations and benefits that constitute an effort/reward bargain between employer and employee [22], even if it also works for describing the relationships between potential employers and job applicants.

Some studies indicate a positive relationship between perceived distributive fairness and selection outcomes, as well as a body of research that has focused on procedural fairness and applicants’ reactions toward the organisation, such as, for example, job acceptance or recommendation intentions [17,23,24,25,26,27,28,29]. 

In term of organisational attractiveness, there is a difference between rejected and accepted applicants: In particular, it appears that for applicants who were hired, fairness of the outcome is more important in influencing post-outcome perceptions; for those who were rejected, procedural fairness is crucial in determining organisational perception after selection [18,30,31]. 

In applicative terms, it seems clear that perceived procedural fairness in the selection process calls for a more sensitive approach by potential employers, in order to implement better external communication standards and procedures and to ensure rejected applicants react in a more positive way. 

## 4. Rejection Procedure and Candidates’ Reactions about the Selection Process

The empirical evidence seems to suggest that variables of different nature affect applicant perceptions of the selection process [11,14,17,18]: The applicant characteristics (e.g., work experience, personality, age) [32], perceptions of procedure characteristics (e.g., transparency, length of process, recruitment media) [33,34,35] and job characteristics (e.g., attractiveness, requirements) influence applicant reactions in terms of fairness and perceived procedural justice, which, in turn, influence applicant outcomes (in term of organisational recommendation, satisfaction, performance) [20], with potential outcomes in terms of future job identity [36]. 

Where the specialised literature has focused on rejection letter characteristics or on rejection procedure, it has highlighted that different factors can influence applicant perceptions and, in particular, fairness perceptions; some studies have focused more on the content of rejection letters, paying specific attention to the effect on candidate reactions of explanations detailing the reasons for rejection (e.g., missing qualifications, rejecting conditions) [8,37,38], and of presence of mitigating sentences and wording (e.g., comment about the applicant’s qualifications, or indirectness of the letter) [6,39,40]. Other research has provided indication on the effect of more procedural characteristics of the communication on applicant perception, such as aspects of customisation of the communication process (personally addressed notification) [37], and time interval [9,41]. 

However, many of these studies were cross-sectional, scenario-based experiments or correlational, and it seems that, overall, they gave insufficient attention to the relationship between a letter’s characteristics, perceived fairness, and outcomes. The purpose of the present study, with a semi-experimental approach and in a context of real selection, is to contribute to the knowledge of this issue and to related communication practices in personnel selection. 

## 5. Confirming the Relationship between Variables Expressed by the Selection Fairness Theory 

The objective of the present research is to explore and understand the effect of operational characteristics (timing, formality level, customization) of the selection results communication on fairness perceptions and on fundamental outcomes (satisfaction, organizational recommendation, intention to reply). More specifically, the paper is interested in understanding the role and the impact of operative characteristics of the feedback communication process on fairness perceptions and selection outcomes; as a propaedeutic goal, the present research aims to verify and confirm literature indications concerning the relationships between fairness perceptions and satisfaction, intention to re-apply, and organizational recommendation expressed by the theoretical reference model [39]. 

Referring to the Selection Fairness Theory [14], according to which perceived fairness experienced during the selection process has an effect on outcomes, the research hypothesized the following:

**Hypothesis** **1 (H1).** *Perceived procedural fairness positively affects organisational recommendations, willingness to re-apply, and satisfaction. More specifically, it was expected that applicants with more positive fairness perceptions of the selection process will be more satisfied and will be more likely to encourage others to apply to the organisation and to re-apply (H1a). Furthermore, the study aims to verify if there are any modifications in candidate perceptions in different selection steps [42]; it is hypothesized that, due to the growth of expectations, the perceptions of the rejected candidates worsen with the progressive overcoming of the selection steps (H1b).*

In order to better clarify the proposed model, the following variables were evaluated as control variables: Gender, age, educational level, and past experience in selection processes. 

Basing on previous literature findings [11,17,32], the study hypothesised the following:

**Hypothesis** **2 (H2).** *Some of the demographic variables can affect perceived fairness and the considered outcomes. In particular, it was expected to find that gender, age, and schooling affect candidate perceptions (H2a). More specifically, it was expected that being used to (or not) selection processes and to receiving notifications may have a role in terms of what applicants expect from organisations. The research hypothesized, finally, that applicants with higher levels of experience will have the best perceptions of the selection process, will be more satisfied and will be more likely to encourage others to apply in the future (H2b).*

## 6. Feedback Communication Characteristics and Candidate Perceptions: A Relationship to Be Clarified

Furthermore, the paper intended to integrate the fairness-based theory with the pragmatics of organizational communication [8,9], considering the rejection letter and procedure as parts of the communication act between a potential employer and an applicant, which needs to effectively manage all the rules and dynamics required by the ongoing interaction. According to the Cooperative Principle [43], if a candidate experiences a violation in this communicative interaction (for example, failure to give feedback or inadequate explanations), she/he will construct meaning and will give a specific intentionality to the potential employer, with a negative impact on corporate image.

### 6.1. Manipulation of the Feedback Communication Process 

In this regard, the present study was designed with 3 different manipulations of the feedback process and of letter characteristics, as factors that can be possibly perceived as a violation of the communication interaction and that are supposed to influence applicant perceptions. Basing on literature findings [37], the following three operational characteristics of the feedback process were manipulated: Response latency of the rejection letter (short latency or long latency), customisation of the communication process (personally addressed notification or impersonal notification), politeness formula (formal or informal). 

Regarding the time latency of the notification letter, a subsample of participants received a response from the selection process two weeks after sending their CV (or two weeks after they were interviewed in the other two steps of the selection), while the other subsample with a two-month response latency. With reference to the manipulation of the politeness formula of the rejection letter, a subsample of participants received a notification letter using a politeness formula to address the applicant (pronoun “Lei”), while the other received a notification letter using the informal formula to address the reader (pronoun “Tu”). Finally, the customisation of the letter was manipulated: A subsample of participants received a personalised notification letter using the candidates’ names (“Dear Applicant’s name”), while the other subsample received a generalised mail (“Dear applicant”). 

### 6.2. The Effect of Manipulating Rejection Process on Candidates’ Perceptions

It seems to us that these modifications of the communication procedures may play an important role in shaping perceived fairness and selection outcomes, with the potential to be perceived as a breach of the interaction between parties. Following the aforementioned rationale, the study proposed the following hypothesises:

**Hypothesis** **3 (H3).** *Response latency in rejection communication (brief vs. long) affects the applicant’s perceptions. It was expected that applicants receiving a brief response latency mail will have better perceptions of the selection procedure fairness (H3a). Moreover, we hypothesized that response latency in rejection communication (brief vs. long) also affects selection outcomes (H3b). Applicants receiving brief response latency letters will be more satisfied and will be more likely to encourage others to apply to the organisation or to re-apply themselves.*

**Hypothesis** **4 (H4).** *The politeness formula in rejection communication (formal vs. informal) affects candidate reactions. Since the Italian language has two different pronouns for the second person singular, namely “tu”, which is used for relatives, friends and, in more general terms, for informal and colloquial conversations, and “lei” which is the polite and formal way to address the second person singular, the study supposed that applicants receiving a more formal response letter will have better fairness perceptions of the selection procedure in comparison to those ones receiving colloquial letters using the “tu” formula (H4a). Besides, the research hypothesized that the politeness formula in rejection communication (formal vs. informal) affects satisfaction, willingness to re-apply and organisational recommendation (H4b). The research supposed that applicants getting a more formal response letter will be more satisfied and will be more likely to encourage others to apply to the organisation or re-apply themselves.*

**Hypothesis** **5 (H5).** *Customisation of rejection communication (customised vs. generalised) affects applicant reactions. We expect that applicants receiving a more customised response letter will have better perceptions of the selection procedure fairness (H5a). In addition, we expect that customisation of rejection communication (customised vs. generalised) affects satisfaction, willingness to re-apply and organisational recommendation (H5b). The study hypothesized that applicants receiving a more customised response letter will be more satisfied and will be more likely to encourage others to apply to the organisation or to re-apply themselves.*

## 7. Method

### 7.1. Participants and Procedure 

Three hundred and twelve candidates, actively seeking employment, applied for a job offer that appeared on the main Italian job search portals for 30 days. The job offer was related to a full-time position in the logistics sector, with the company initially offering a fixed-term contract (12 months), leading to possible permanent employment. The recruitment process included 3 selection steps (Step 1: CV Screening; Step 2: Phone Interview; Step 3: Individual Interview) and was entirely managed by the HR office of a medium-sized Italian company operating in the logistics sector. Candidates rejected in the first phase were randomly assigned one of the 8 (2 x 2 x 2) conditions, and then received a specific notification email, differing for each condition. The rejection notifications were manipulated in 3 ways: the response latency of the notification letter (2 weeks vs. 2 months), the politeness formula of the letter (formal vs. informal), and the customisation of the letter (anonymous vs. personalised with the candidate’s name). Within three days of receiving the rejection letter, applicants were asked by email to complete an online anonymous self-administered questionnaire. There were four possible rejection letters, sent with two different time latencies (brief and long): Formal and Customised, Formal and Anonymous, Informal and Customised, Informal and Anonymous. An example of the content of the letter is presented below. 

Rejection letter example: Dear ‘Name of the candidate’/Dear candidate, it was really a pleasure to meet you and evaluate your professional qualities and experience. As coordinator of the selection process, I inform you that the company, while considering your profile and skills to be of certain interest, is turning its attention to other candidates for the next step of the selection process. We thank you for the interest and the time you wanted to dedicate to us, and we wish you the best in your future job search. 

The sample considered consisted of 142 participants rejected at Step 1 of selection (CV screening), who replied to the survey (response rate = 49%). The sample (77 males and 65 females) presented an average age of 35.5 years (SD = 7.8), and a medium–high education level (Diploma, n = 78, 54.9%; 3-year degree, n = 21, 14.7%; Master’s degree, n = 32, 22.5%; post-degree specialisation, n = 11, 7.9%). Samples of other candidates who reached Step 2 (n = 58) and Step 3 of the selection (n = 15), who replied to the survey (Step 2 response rate = 41%, n = 24; Step 3 response rate = 26%, n = 4), due to the low number of participants in many of the 8 experimental conditions, and given the consequent impossibility of an appropriate comparison, have been excluded from statistical analysis. The final sample of analysis was 142 candidates rejected at Step 1 of selection. 

### 7.2. Design

The design was a 2 × 2 × 2 factorial plan with a response latency of the notification letter (two weeks vs. two months), a politeness formula of the letter (formal vs. informal), and customisation of the letter (anonymous vs. personalised with the candidate’s name) as between-subjects variables. With the aim of testing hypotheses, correlation and regression analyses with SPSS 21.0 (IBM, Armonk, NY, USA), were conducted. Moreover, we tested the proposed model through SEM using SPSS AMOS 22 (IBM, Armonk, NY, USA). Lastly, the possible role of manipulation of the feedback process on perceptions was explored through ANOVA and moderated mediation analysis with SPSS PROCESS (IBM, Armonk, NY, USA) (Dawson, 2013). 

### 7.3. Measures

The questionnaire was made up of an introductory section to collect socio-demographic data (gender, age, nationality, educational level, employment status) and assessed active participation in selection processes as candidate selection seniority (number of applications sent and number of notification letters received in the last three months) and a section made up of the scales that will be described in the following section. The company, a partner of the research, recommended and allowed the use of a questionnaire that should not have involved the candidates for more than five minutes, with limited questions (max 20 items). Moreover, because of concern with the response rate critical issue in the selection context, we decided to use many single-items measures, with the intention of being easily understood by job applicants of all social backgrounds and schooling, and of making a brief but valid questionnaire [44,45]. Statistical techniques to address the inability to determine internal consistency reliability for the single-item of job satisfaction, which is considered the main weakness of a single-item measure, demonstrate promising results [46]. Using a single questionnaire for all variables with many single-item measures, we tried to limit common method variance according to methods outlined in the literature [47]. We randomly inserted the different scales into the questionnaire and they were graphically separated from each other. Different scale endpoints and formats for the measures were used in order to reduce method biases caused by commonalities in scale endpoints and anchoring effects. 

#### 7.3.1. Perceived Procedural Fairness

Perceived fairness and equity of the selection process were measured with five items (example “I believe that the evaluation was objective and impartial”, “I believe that this selection treated all the candidates in the same way”), on a five-point Likert-scale, ranging from 1 (strongly disagree) to 5 (strongly agree). The scale was derived from the international literature [24,38]. Cronbach’s Alpha value of the scale was excellent (alpha = 0.955).

#### 7.3.2. Organisational Recommendation

Basing on the suggestion and measure developed by Waung and Brice [12], overall organisational recommendation was measured with one item assessing the likelihood of encouraging others to apply to the organisation (“Would you recommend an application for this company to a friend?”), on a four-point Likert-scale, ranging from 0 (I would certainly not recommend this company) to 3 (I would certainly recommend this company). 

##### Willingness to Apply Again

Willingness to apply again was measured with one item assessing the likelihood of considering a new selection process in case of a new vacant position in the company (“Would you apply again to this company?”), on a five-point Likert-scale, ranging from 0 (I will certainly not re-apply) to 4 (I will certainly re-apply). 

#### 7.3.3. Satisfaction

Satisfaction with the selection process was measured with a single item (“How satisfied are you with the selection process?”) on a four-point Likert-scale, ranging from 1 (totally dissatisfied) to 4 (totally satisfied). 

#### 7.3.4. Past Experiences

Past experience in selection processes, and namely the extent to which applicants are used to receiving rejection letters, were measured by two different items, (a) one assessing the number of CVs sent during the last three months (“Referring to the last three months, how many CVs did you submit to apply for job searches?”) on a four-point Likert-scale, ranging from 1 (0–5 CVs) to 4 (more than 15); (b) one assessing the number of responses (notification letters) that applicants received in the last three months (“Referring to the last three months, how many notification letter did you receive in response?”) on a four-point Likert-scale, ranging from 1 (none) to 5 (one for each application).

## 8. Results

Preliminary analyses were performed to ensure there was no violation of the assumption of normality, linearity, and multicollinearity (descriptive statistics in Table 1 and Table 2). Multi-variate outliers were identified thanks to the Mahalanobis Distance and the sample was reduced to 138 rejected applicants, in line with the exploratory nature of the study.

### 8.1. Fairness Perceptions Effect on Outcomes

**Figure 1:**
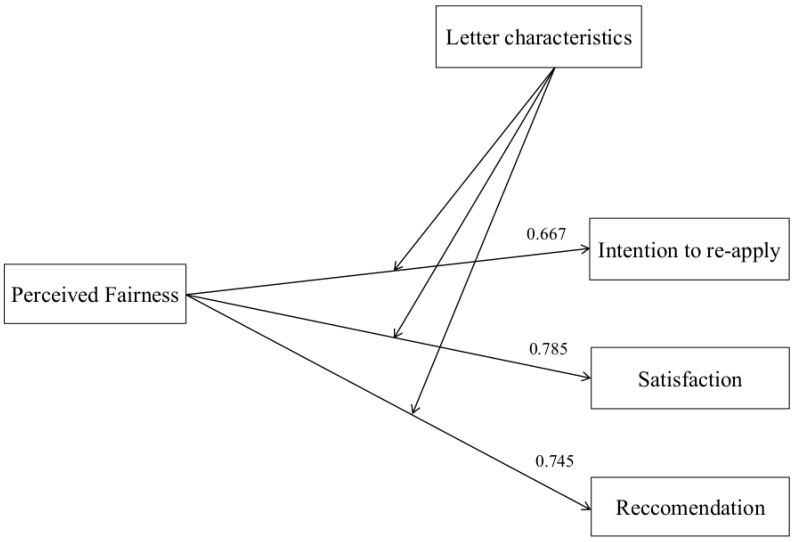
Input Path Diagram of the proposed theoretical model

In order to verify Hypothesis 1 and confirm the relation between fairness perceptions and outcomes, as expressed by the reference theoretical model [14], a regression analysis was conducted with fairness perception as independent variable: Perceived fairness of the selection process positively and strongly predicts organisational recommendation (*R*^2^ = 0.555, *AdjR*^2^ = 0.552; *F* (1, 137) = 174,451, *p* < 0.001; *B* = 0.859, *β* = 0.745, *t* = 13.208, *p* < 0.001), willingness to re-apply (*R*^2^ = 0.445, *AdjR*^2^ = 0.441; *F* (1, 137) = 112, 44, *p* < 0.001, *B* = 0.761, *β* = 0.667, *t* = 10.6, *p* < 0.001) and satisfaction (*R*^2^ = 0.616; *AdjR*^2^ = 0.613; *F* (1, 137) = 224,441; *p* < 0.001; *B* = 0.967, *β* = 0.785, *t* = 14.981, *p* < 0.001). The hypotheses concerning relationships between variables were investigated through structural equation modelling (SEM). Five fit indices were used: The chi square test, the comparative fit index (CFI), the goodness-of-fit statistic (GFI), the normed-fit index (NFI), the root mean-square error of approximation (RMSEA), and the standardized root mean square residual (SRMR). Basing on our theoretical foundation, we specified a model in which fairness perceptions predicts all the outcomes (Figure 1). This model showed acceptable fit, χ² (df = 3) = 20.985, *p* < 0.001; CFI = 0.948, GFI = 0.956; NFI = 0.941; RMSEA = 0.011; SRMR = 0.012. Overall, these results seem to clearly confirm Hypothesis 1. 

Lastly, given the low relative number of participants in the semi-experimental conditions of Step 2 and 3 of the selection, the study did not proceed to test the sub-Hypothesis 1b (candidate perception differences between selection steps). The only datum worthy of comment concerns the dramatic drop in the response rate from Step 1 (46%) to Step 3 (26%), which could be, however, be interpreted as a progressive collapse of the motivation to participate in the research, due to an increase in expectations as an applicant proceeds in the selection.

### 8.2. Socio-Demographic Factor Effects on Candidate Perceptions 

With the aim of exploring the effect of demographic factors on candidates’ reactions, a *t*-test and one-way ANOVA for gender and schooling, and linear regression for level of education, were conducted for all the variables. No significant relationship was found between demographic variables (age, gender, education) and perceptions of the selection process or outcomes; results, consequently, did not support Hypothesis 2a. Regarding past experiences in rejection letters and selection processes, results showed no relationship with fairness perceptions. Moreover, experience did not predict any outcomes except for a negative prediction of organisational recommendation (frequency of applying for vacant position: *R*^2^ = 0.027, *AdjR*^2^ = 0.20, *F* (1, 137) = 3,692; *B* = −0.208, *β* = −0.161, *t* = −0.1.921, *p* < 0.05): results, unexpectedly, underlined that the less candidates have applied for vacant positions, the more they will recommend the company to other people. Contrary to what was expected, Hp2b was not confirmed and was, indeed, overturned.

**Figure 2:**
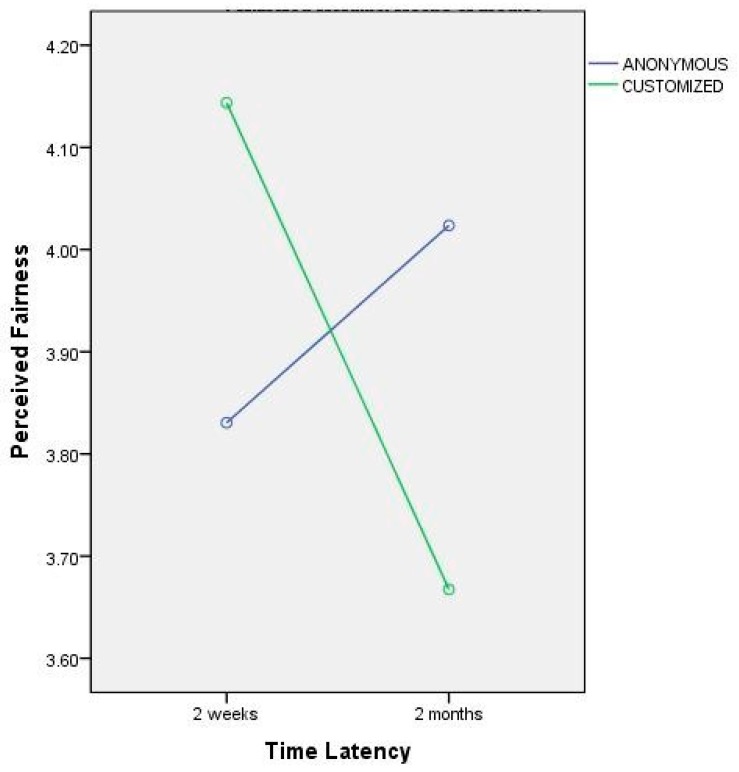
Interaction effect between time latency and customisation of rejection letter on perceived fairness.

In order to test if manipulations of the feedback process would be perceived as a violation of the communication interaction, a 2 x 2 x 2 mixed-model ANOVA was used to test remaining hypotheses (H3–5), with the customisation, politeness formula, and time latency of the notification letter as between-subjects factors. Results showed no main effect of the three factors on fairness perceptions, except for an interaction between time latency and customisation (*F* = 3.39, *p* < 0.50; Figure 2), and an interaction between all the three factors (*F* = 3.9, *p* < 0.050; Figure 2). 

When the time latency of the rejection letter is short, if the communication is personalised with the name of the applicant, candidates have higher fairness perceptions compared to those who received an anonymous letter; when the time latency of the letter is longer, if the communication is personalised with the name of the applicant, candidates have lower fairness perceptions compared to those who received an anonymous letter (Figure 2). 

**Figure 3:**
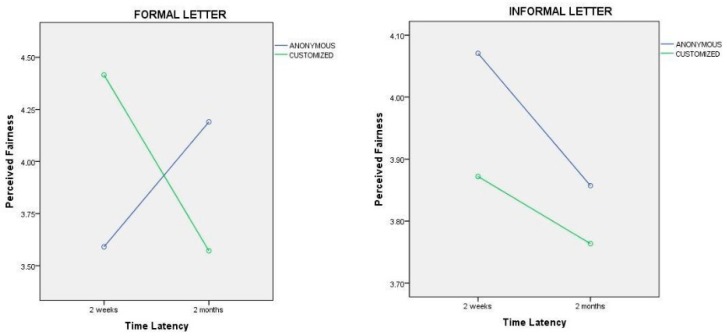
Interaction effect between time latency, customisation and politeness formula of the letter on fairness perceptions.

The same interaction is confirmed for formal letters, while it is not confirmed for informal letters: When the rejection communication is anonymous and written in an informal way, no matter what the time latency, candidates have higher fairness perceptions compared to those who received a personalised letter (Figure 3). H3a, H4a, and H5a regarding the effect of feedback characteristics on fairness perceptions seem not to be confirmed by the results, and some of the hypothesized consequences of experimental manipulation were somehow subverted. 

ANOVA showed a significant effect of the response latency (*F* (1, 137) = 3.890; *p* < 0.05) and of the politeness formula (*F* (1, 140) = 4.187, *p* < 0.05) on the willingness to re-apply: Candidates who received a faster response showed a higher propensity to re-apply for a vacant position with the same organisation (short latency = 4.1, SD = 1.17; long latency = 3.89, SD = 1.29); furthermore, candidates who received informal letters showed higher propensity to re-apply for a vacant position with the same organisation compared to those who received a formal rejection letter (informal = 4.06, SD = 1.31; formal = 3.87, SD = 1.17). ANOVA revealed no other main effect of the three manipulations on organisational Recommendation and willingness to re-apply, except for an interaction among all the three (on organisational recommendation: *F* = 3.27, *p* < 0.050; on willingness to re-apply: *F* = 3.22, *p* < 0.50). Manipulation of selection communication processes seem to especially affect, between selection outcomes, willingness to re-apply; these findings appear to confirm partially H3b, H4b, and H5b. Results disclosed no main effect nor interaction of the three factors on satisfaction with the selection process. 

### 8.3. The Role of Feedback Process Characteristics on Selection Perceptions: Additional Analysis

Because of the lack of direct effects of the manipulated characteristics of the rejection letters on many perceptions, and in order to better specify and explore the role of feedback process manipulation on the measured variables, as a post hoc analysis of the data, the research tested all the possible mediation between perceived fairness and each of the outcome for all the three manipulations. A Moderated Mediation Analysis with the aid of SPSS PROCESS Macro software was performed (Figure 1) [48].

Results showed that only the time latency of the refusal letter can be considered a moderator of the relation between perceived fairness and willingness to re-apply (*R*^2^ = 0.6795, *AdjR*^2^ = 0.4617, *F* (3, 138) = 39.45, *p* < 0.001; *R^2^-change* = 0.0156, *F* (1, 138) = 3.999, *p* < 0.05). No other moderation effect was found for any of the other variables. 

## 9. Discussion and Conclusions

The job market is characterised by strong competition between candidates, while the concept of career has lost the connotations of an organisational process and has become a path managed entirely by the individual, conditioned daily by occupational circumstances requiring greater flexibility from the worker and the ability to handle multiple identities and roles [49]. The pervasive and sudden transformation of the work scene has led to a major review of personnel management strategies, talent-attracting and selection practices, and a parallel change in worker perceptions, emotional content and cognition [50]. Technology has led to the diffusion of selection tools and instruments that help with recruitment strategies but sometimes create impersonal recruitment processes, especially from a communication point of view. Overall, while technology seems to help organisations to receive hundreds of applications, communication of the selection results is not always adequately managed [25]. 

Adopting a semi-experimental approach, the study tried to go beyond the mainly scenario-based research on applicant reactions, comparing the effect of different manipulations of the rejection feedback procedure on perceptions. 

First of all, the results endorsed the extreme complexity of doing research in real selection contexts: Operational needs, standards and time availability in companies and employment agencies are often difficult to reconcile with scientific rigour and methodologies; moreover, taking into account candidate response rate, it seems that these critical issues increase with the progress of the selection process. 

Overall, the results contributed to literature confirming that fairness perceptions of the selection process strongly relate and predict selection outcomes (organisational recommendation, intention to re-apply, and satisfaction), supporting Gilliland’s theoretical model [38,41]. Furthermore, the influence of socio-demographic factors on candidate reaction was not demonstrated; only the frequency of applying was found to negatively predict organisational recommendation: This seems to indicate that the role of past experience in selection processes can possibly worsen an applicant’s perception of the selection process, or, more simply, that the frequency of applying is influenced by other personal variables (expectation, motivation, employability, etc.) that will have to be investigated in the future. 

The influence of the characteristics of the rejection process on perceptions has not been fully clarified by our data. Certainly, willingness to re-apply and fairness perceptions seem the most sensitive factors to changes in refusal procedures, hence manipulations will certainly have to be improved. 

Some of the characteristics of the rejection letter (time latency and politeness formula) showed an effect on the willingness to re-apply: As expected, receiving a notification with a long delay left the candidate less likely to re-apply; but unexpectedly, receiving an informal letter seems to be perceived in a more positive way, with candidates showing a higher willingness to re-apply compared to those who received formal rejections. Moreover, even an anonymous refusal seems to be better accepted in terms of fairness, compared to a personalized letter. 

The reason for this result may be that formal communication possibly increases distance between interlocutors; in other words, being addressed in an informal and anonymous way may lead the rejected candidate to feel closer to the rejecting employer and to accept more easily the negative communication 

Overall, results seem to suggest that applicants prefer a fast feedback with an informal style, while the effect of the customization of the letter must be cleared by further studies. The interactions highlighted among the characteristics of the rejection process can be explained by the possible differential effect on perception of the latency of the letter: Feedback with the right time latency is considered to be preferred, informal and impersonal, whereas when receiving feedback with extreme delay, these characteristics should be perceived as a further violation of the communication process. The time latency of the rejection letter seems to play more a moderating role between fairness perceptions and outcomes, rather than being an antecedent. 

### 9.1. Limitations of the Study

The present research has many limitations; the size of the sample represents a problem in terms of result generalisation, significance of the effects, and internal validity; this needs to be taken into account and future research may, therefore, focus on confirming the pattern of relationships found within a larger sample. Limitations can certainly be identified in some of the choices of manipulating the rejection communication process: For example, the decision to create a condition in which candidates receive the refusal notification in two months if, on one hand, wanted to create a situation of clear violation of the communication, it could have produced difficulty for the candidates to remember which specific selection process they had participated in. Consequently, it’s mandatory to develop further studies to investigate the exact role played by rejection procedures on perceptions, identifying new effective and plausible manipulations. Another limitation is the use of a self-report questionnaire for the data collection, with many single-item measures, and few variables measured, that limits construct validity. Data collection through a self-report questionnaire could have inflated common method variance bias, although we adopted several strategies suggested by the literature in order to, at least partially, counterbalance such bias. In particular, administration through electronic survey might have influenced those who might be unfamiliar with such an interface differently than for those who might be familiar. Inter-correlations among measures affected by CMV could have been inflated or deflated depending upon many factors [51]. 

### 9.2. Practical Implications and Future Research Agenda

In conclusion, our results suggest that additional investment should be made in organisational communication, considering that providing feedback is a relatively low-cost activity which has a high impact on job applicants [52]. Furthermore, those who are involved in the hiring process should be trained on how to communicate feedback, especially that of a negative nature [38,53,54]. Finally, in consideration of the current study findings, we suggest an avenue for future research that advances a greater understanding of the processes of organisational communication during personnel selection. In particular, these recommendations include further investigation of the different dispositional variables that may be moderators and/or mediators between communication procedure and candidate reactions. 

### 9.3. Conclusions 

The agenda for future research should also involve qualitative studies on the experience of selection rejection, considering additional new variables, such as generational effects and the different selection steps during which rejection occurs, along with “real” customisation of letters (giving reasons for the decision). An additional issue to be addressed is the role played by third party employment branding, which could affect applicant reactions [55] and the electronic word-of-mouth spread by rejected candidates [56] and that played by social media reputation [57,58,59] also due to the heightened sensitivity of candidates during the selection time period. Organisations should be sensitive to the issue of refusal communication to support their employer branding; proper attention should be given to the research and development of communication processes and protocols that are able to relay negative information without affecting candidate self-esteem [60]. Rejection letters are organisational communication tools that can help to promote corporate image, and that can encourage others to think positively about the organisation and to consider it as a possible choice of employer for future opportunities [17,37].

## Figures and Tables

**Table 1:** Descriptive statistics for all the measured variables.

	Min	Max	Mean	SD	Skewness	Kurtosis
**Number of CVs in the last three months**	1	4	3.24	1.01	−0.963	−0.485
**Number of received notifications in the last three months**	1	5	3.69	3.30	1.085	0.378
**Perceived fairness**	1	5	3.89	1.13	0.936	0.569
**Organizational recommendation**	1	4	3.41	1.3	−0.365	−0.940
**Willingness to re-apply**	1	5	3.99	1.29	−0.991	0.094
**Satisfaction**	1	4	3.24	1.39	−0.244	−0.947

**Table 2:** Zero-order correlations for all the measured variables.

	1	2	3	4	5	6
**1 Number of CVs in the last three months**	1					
**2 Number of received notifications in the last three months**	0.218 **	1				
**3 Perceived fairness**	−0.1	0.132	1			
**4 Organizational recommendation**	−0.161	0.140	0.745 ***	1		
**5 Willingness to re-apply**	−0.073	0.076	0.667 ***	0.641 ***	1	
**6 Satisfaction**	−0.090	0.155	0.785 ***	0.675 ***	0.600 ***	1

** = *p* < 0.01; *** = *p* < 0.001.
